# The PREVENT dementia programme: baseline demographic, lifestyle, imaging and cognitive data from a midlife cohort study investigating risk factors for dementia

**DOI:** 10.1093/braincomms/fcae189

**Published:** 2024-05-31

**Authors:** Craig W Ritchie, Katie Bridgeman, Sarah Gregory, John T O’Brien, Samuel O Danso, Maria-Eleni Dounavi, Isabelle Carriere, David Driscoll, Robert Hillary, Ivan Koychev, Brian Lawlor, Lorina Naci, Li Su, Audrey Low, Elijah Mak, Paresh Malhotra, Jean Manson, Riccardo Marioni, Lee Murphy, Georgios Ntailianis, William Stewart, Graciela Muniz-Terrera, Karen Ritchie

**Affiliations:** Edinburgh Dementia Prevention, Centre for Clinical Brain Sciences, University of Edinburgh, Edinburgh, EH4 2XU, UK; Scottish Brain Sciences, Edinburgh, EH12 9DQ, UK; School of Medicine, University of St Andrews, St Andrews, KY16 9TF, UK; Edinburgh Dementia Prevention, Centre for Clinical Brain Sciences, University of Edinburgh, Edinburgh, EH4 2XU, UK; Edinburgh Dementia Prevention, Centre for Clinical Brain Sciences, University of Edinburgh, Edinburgh, EH4 2XU, UK; Scottish Brain Sciences, Edinburgh, EH12 9DQ, UK; Department of Psychiatry, School of Clinical Medicine, University of Cambridge, Cambridge, CB2 2QQ, UK; Edinburgh Dementia Prevention, Centre for Clinical Brain Sciences, University of Edinburgh, Edinburgh, EH4 2XU, UK; Department of Psychiatry, School of Clinical Medicine, University of Cambridge, Cambridge, CB2 2QQ, UK; INM, Université de Montpellier, INSERM, Montpellier, 34091, France; PREVENT Participants Panel Member, London, UK; Edinburgh Dementia Prevention, Centre for Clinical Brain Sciences, University of Edinburgh, Edinburgh, EH4 2XU, UK; Department of Psychiatry, University of Oxford, Oxford, OX3 7JX, UK; Global Brain Health Institute, Trinity College Dublin, Dublin 2, Ireland; Global Brain Health Institute, Trinity College Dublin, Dublin 2, Ireland; Trinity College Institute of Neuroscience, School of Psychology, Trinity College Dublin, Dublin, D02 PX31, Ireland; Department of Psychiatry, School of Clinical Medicine, University of Cambridge, Cambridge, CB2 2QQ, UK; Department of Neuroscience, University of Sheffield, Sheffield, S10 2HQ, UK; Department of Psychiatry, School of Clinical Medicine, University of Cambridge, Cambridge, CB2 2QQ, UK; Department of Psychiatry, School of Clinical Medicine, University of Cambridge, Cambridge, CB2 2QQ, UK; Imperial College London, UK Dementia Research Institute Care Research and Technology Centre, London, W12 0BZ, UK; Brain Sciences, Imperial College London, London, W12 0NN, UK; Clinical Neurosciences, Imperial College Healthcare NHS Trust, Charing Cross Hospital, London, W6 8RF, UK; Edinburgh Dementia Prevention, Centre for Clinical Brain Sciences, University of Edinburgh, Edinburgh, EH4 2XU, UK; Roslin Institute, University of Edinburgh, Edinburgh, EH25 9RG, UK; Centre for Genomic and Experimental Medicine, Institute of Genetics and Cancer, University of Edinburgh, Edinburgh, EH4 2XU, UK; Edinburgh Clinical Research Facility, University of Edinburgh, Edinburgh, EH4 2XU, UK; Edinburgh Dementia Prevention, Centre for Clinical Brain Sciences, University of Edinburgh, Edinburgh, EH4 2XU, UK; Department of Neuropathology, Queen Elizabeth University Hospital, Glasgow, G51 4TF, UK; School of Psychology and Neuroscience, University of Glasgow, Glasgow, G12 8QB, UK; Edinburgh Dementia Prevention, Centre for Clinical Brain Sciences, University of Edinburgh, Edinburgh, EH4 2XU, UK; Ohio University Heritage College of Osteopathic Medicine, Ohio University, Ohio, OH 45701, USA; Edinburgh Dementia Prevention, Centre for Clinical Brain Sciences, University of Edinburgh, Edinburgh, EH4 2XU, UK; INM, Université de Montpellier, INSERM, Montpellier, 34091, France

**Keywords:** observational cohort, brain health, Alzheimer’s disease, preclinical dementia, risk factors

## Abstract

PREVENT is a multi-centre prospective cohort study in the UK and Ireland that aims to examine midlife risk factors for dementia and identify and describe the earliest indices of disease development. The PREVENT dementia programme is one of the original epidemiological initiatives targeting midlife as a critical window for intervention in neurodegenerative conditions. This paper provides an overview of the study protocol and presents the first summary results from the initial baseline data to describe the cohort. Participants in the PREVENT cohort provide demographic data, biological samples (blood, saliva, urine and optional cerebrospinal fluid), lifestyle and psychological questionnaires, undergo a comprehensive cognitive test battery and are imaged using multi-modal 3-T MRI scanning, with both structural and functional sequences. The PREVENT cohort governance structure is described, which includes a steering committee, a scientific advisory board and core patient and public involvement groups. A number of sub-studies that supplement the main PREVENT cohort are also described. The PREVENT cohort baseline data include 700 participants recruited between 2014 and 2020 across five sites in the UK and Ireland (Cambridge, Dublin, Edinburgh, London and Oxford). At baseline, participants had a mean age of 51.2 years (range 40–59, SD ± 5.47), with the majority female (*n* = 433, 61.9%). There was a near equal distribution of participants with and without a parental history of dementia (51.4% versus 48.6%) and a relatively high prevalence of APOEɛ4 carriers (*n* = 264, 38.0%). Participants were highly educated (16.7 ± 3.44 years of education), were mainly of European Ancestry (*n* = 672, 95.9%) and were cognitively healthy as measured by the Addenbrookes Cognitive Examination-III (total score 95.6 ± 4.06). Mean white matter hyperintensity volume at recruitment was 2.26 ± 2.77 ml (median = 1.39 ml), with hippocampal volume being 8.15 ± 0.79 ml. There was good representation of known dementia risk factors in the cohort. The PREVENT cohort offers a novel data set to explore midlife risk factors and early signs of neurodegenerative disease. Data are available open access at no cost via the Alzheimer’s Disease Data Initiative platform and Dementia Platforms UK platform pending approval of the data access request from the PREVENT steering group committee.

## Teaser

PREVENT is a multi-centre prospective cohort study with 700 participants aged 40–59 from the UK and Ireland that aims to examine midlife risk factors for dementia. This paper provides an overview of the study protocol and presents the first summary results from the initial baseline data to describe the cohort.

## Graphical Abstract

**Graphical Abstract:**
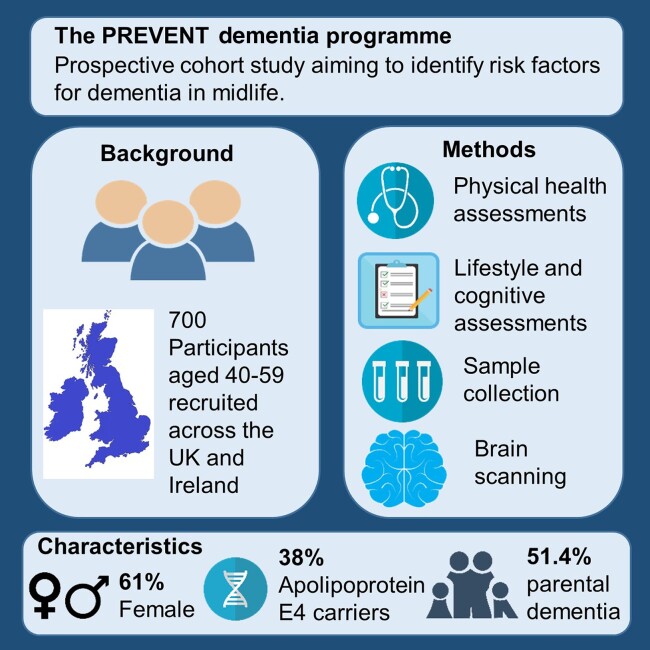

## Introduction

The PREVENT dementia programme was initiated in 2014 as a single-site study based in West London. It has subsequently expanded to become a multi-centre study, opening sites in Edinburgh (2015), Oxford (2017), Cambridge (2017) and Dublin (2018). The aims of PREVENT are to profile midlife risk factors for later-life neurodegeneration and to identify the earliest indices heralding neurodegenerative disease in advance of clinically diagnosable dementia (particularly Alzheimer’s disease). The original baseline protocol for the pilot site is described elsewhere,^1,2^ with this current paper serving to provide an update on the protocol, detailing a multitude of sub-studies supplementing the main study, and provide an overview of the baseline data set.

## Recruitment

Participants aged 40–59 years old at the time of consent were recruited to the study providing they did not already have a diagnosis of dementia or a known contraindication to having an MRI scan. Their cognitive status was assessed through the Addenbrookes Cognitive Examination 3 (ACE-III), and although this did not occur, they would have been withdrawn if they scored below the appropriate scores set dependent on an individual’s age.

Various recruitment methods were used to recruit the participants across all sites. Initially, participants were recruited as family members of patients at National Health Service (NHS) memory clinics at the participating sites and through local dementia research registers. Following this, family and friends of participants were invited to participate and recruitment took place via word of mouth. The Join Dementia Research platform (www.joindementiaresearch.nihr.ac.uk) was also utilized to recruit participants (at all sites except Trinity College Dublin) along with some participants registering their interest to participate through the PREVENT dementia website (www.preventdementia.co.uk). No geographic limitations were placed on recruitment; participants were eligible to attend any site if able to travel and complete all protocol assessments.

Across the five centres, 700 participants have completed baseline assessments and the first follow-up (Visit 2) around 2 years after baseline. A second wave of follow-up visits is underway at the London site and planned at the other centres, re-assessing participants at 5 to 8 years post-baseline.

PREVENT has also collaborated with a number of sister projects since its inception. The TriBEKa collaboration (https://www.barcelonabeta.org/en/research/research-studies/tribeka) was established in 2017 between the Barcelona Beta Brain Research centre (the ALFA project^3^), the University of Edinburgh (PREVENT) and the Karolinska Institute, with the aim of supporting ongoing cohorts of healthy adults at a spectrum of risk for dementia with a focus on neuroimaging data collection. The aim of the collaboration is to harmonize neuroimaging data sets where appropriate and support the addition of rich neuroimaging data from the cohorts to the Global Alzheimer's Association Interactive Network and Alzheimer’s Disease Data Initiative (ADDI) portals for worldwide academic access. In addition to TriBEKa, PREVENT was associated with the European Prevention of Alzheimer’s Dementia (EPAD) programme.^4-6^ The EPAD Longitudinal Cohort Study included a wide spectrum of participants at differing levels of risk for Alzheimer’s disease. In addition to being a recruitment source as a parent cohort, PREVENT influenced the design of the EPAD LCS protocol. Importantly, the participant involvement experience from PREVENT ensured this became a core pillar of EPAD, with a significant impact on the study success reported.^7^ Focus groups involving PREVENT participants also explored ethical aspects of the EPAD project before initiation, which was developed into a work package focusing on ethics within the EPAD project.^8,9^

## Materials and methods: the PREVENT dementia protocol

In this paper, the PREVENT dementia baseline data are described and distributions of key risk and outcome variables relevant to brain health as per the pre-defined three risk groups of low (*APOEɛ4*− and FH−), mid (one of *APOEɛ4*+ or FH+) and high (*APOEɛ4*+ and FH+) are explored comparing these variables between these groups. More specific analysis based on specific hypotheses has formed and will form the basis of other academic outputs.

### Ethics

Multi-site ethical approval was granted by the UK London-Camberwell St Giles NHS Research Ethics Committee (REC reference: 12/LO/1023, IRAS project ID: 88938), which operates according to the Helsinki Declaration of 1975 (and as revised in 1983). A separate ethical application for Ireland was submitted for the Dublin site and was reviewed and given a favourable opinion by Trinity College Dublin School of Psychology Research Ethics Committee (SPREC022021-010) and the St James Hospital/Tallaght University Hospital Joint Research Ethics Committee. All substantial protocol amendments have been reviewed by the same ethics committees, and favourable opinion was granted before implementation at sites. All sub-studies referred to have individual ethical applications and favourable opinions.

### Demographics

Participants self-reported demographic information via an interview with a researcher during each study visit. Demographic data were gathered to provide descriptive data on the cohort and to include a number of known risk and confounding factors for neurodegeneration. The demographic data include the date of birth, sex, years of education, family history of dementia (including subtype, age of onset and age of death where known), occupation, postcode and handedness.

### Biosamples

All participants were asked to provide blood, urine and saliva samples, with an option to undergo a lumbar puncture for cerebrospinal fluid. Approximately 50 ml of blood were collected from overnight fasted participants. Clinical samples were analysed immediately for standard biochemistry and haematology measures at local laboratories, with results entered into the participant database. Research samples were processed and prepared for long-term storage as plasma, buffy coat, serum and whole blood samples (for DNA extraction) and stored at −80°C.

Saliva samples were also collected from all participants on two different days across eight time points. The first day of sample collection, termed a controlled stress day, was the day of their study visit when the clinical and cognitive assessments were completed. Participants were asked to complete the second day of samples (requested to be within a week of the first day of sampling but up to 1 month from the first sample day) on a quieter day at home (quieter day recommended to be a day spent mainly at home where participants did not envisage encountering any significant stressors). Stimulated saliva was collected using Salivette® tubes (Sarstedt, Germany) and cortisol collection tubes with a synthetic swab. Samples were returned to the research unit after completion and stored at −20°C.

A 12-h overnight urine collection was also completed by all participants, which was then processed and prepared for long-term storage. Forty millilitres of urine was extracted and stored for each participant, 20 ml as standard and 20 ml acidified with hydrochloric acid and then stored at −80°C.

All processed biosamples are stored at the Scottish Brain Health Bioresource, The Roslin Institute, University of Edinburgh.

### Genetic data

Genomic DNA from PREVENT participants was isolated from whole blood samples using a Nucleon Kit (Gen-Probe) with the BACC3 protocol. DNA samples were re-suspended in 1-ml TE buffer pH 7.5 (10 mM Tris-Cl pH 7.5 and 1 mM EDTA pH 8.0). The yield of the DNA was measured using picogreen. *APOE* genotyping was performed using TaqMan polymerase chain reaction genotyping and the QuantStudio 12K Flex system (*n* = 696). The final volume was 5 μl using 20 ng of genomic DNA, 2.5 μl of TaqMan Master Mix and 0.125 μl of 40× Assay by Design or 0.25 μl of 20× Assay on Demand Genotyping Assay. The cycling parameters were 95° for 10 min, 40 denaturation cycles at 92° for 15 s and annealing/extension at 60° for 1 min.

Six hundred and ninety-six samples underwent genome-wide genotyping on the Infinium™ Global Screening Array-24 v3.0 BeadChip (*n* = 730 059 loci) and scanned on an Illumina iScan platform. Genotypes were called automatically using GenomeStudio Analysis software v2011.1, and quality control was performed using PLINK v1.9.^10^ Samples and probes were removed based on the following criteria: genotype call rate (<95%), Single nucleotide polymorphisms (SNP) missingness (>1%, --geno 0.01), sample missingness (>1%, --mind 0.01), Hardy–Weinberg equilibrium (*P* < 1 × 10^−6^, --hwe 1e-6), minor allele frequency (<0.5%, --MAF 0.005) and heterozygosity outlying values (*F* statistic > 3 SDs). In total, 647 samples and 515 602 SNPs passed quality control. We further identified and removed 31 individuals related to another cohort member. To protect against sex imbalance in the sample, the first exclusion criterion was to remove females from male–female pairs. The second criterion was to exclude the individual with the poorer genotype call rate in male–male or female–female pairings. We also removed 20 ancestry outliers (i.e. of non-European ancestry), leaving 596 samples in our most stringent data set. Relatedness was estimated via an identity-by-descent coefficient ≥ 0.1875, which represents the halfway point between second- and third-degree relatives. Ancestry outliers were identified by principal component analyses on the PREVENT genotype data set merged with HapMap III reference data. PREVENT genotypes were also imputed against European sample data from the Haplotype Reference Consortium build release 1.1 (GRCh37/hg19), 1000 Genomes Phase 3 (version 5) and the TOPMed r2 reference panel.^11-13^ There were 8 651 773, 9 803 244 and 10 082 029 imputed, autosomal SNPs for the Haplotype Reference Consortium, 1000G and TOPMed panels, respectively (imputation quality score *R*^2^ ≥ 0.6 and minor allle frequency (MAF) ≥ 0.005).

### Physical examination

As part of the clinical assessment participants underwent a physical and neurological examination, an ECG, spirometry (removed during the Covid-19 pandemic), vital signs and anthropometric measurements (height, weight, leg length, waist, hip and neck measurements).

### Imaging

**Figure 1:**
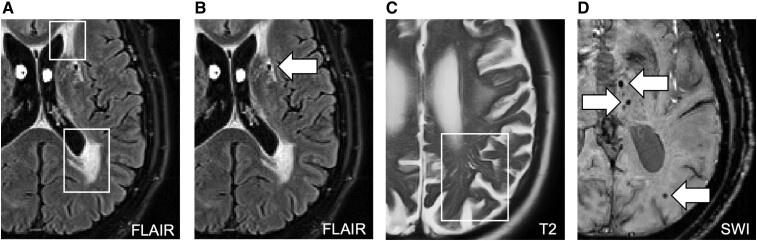
Imaging markers of cerebral small vessel disease taken from Low *et al*.^20^ with permission. Panels reproduced represent: A: white matter hyperintensities; B: lacunes; C: enlarged perivascular spaces; D: cerebral microbleeds.

Six hundred and sixty-six brain imaging data sets were collected using 3-T Siemens MRI scanners (specific models: Verio, PRISMA, Prisma Fit, Skyra). Image processing of the T_1_-weighted structural scans was carried out using FreeSurfer version 7.1.0 following correction for field inhomogeneities using the N4 algorithm.^14,15^ In particular, using the *recon-all* pipeline, global volumetrics, cortical thickness and hippocampal volume were measured. Manual corrections were conservatively applied to the *recon-all* outputs where appropriate by trained operators. Structural MRI scans were also used for the quantification of cerebral small vessel disease markers. White matter hyperintensity (WMH) volumes were quantified from lesion masks obtained from FLAIR MRI using an automated script on SPM8. Lesion maps obtained from the segmentation procedure were used as starting points for manual WMH delineation. WMH volumes were normalized by total intracranial volume to account for differences in head sizes and cube-root transformed, in that order. Non-normalized WMH volumes also underwent cube-root transformation for sensitivity analysis. Details on the procedures involved on all volumetric analyses have been described previously.^16-19^ The diffusion-weighted imaging data sets were first carefully examined for sufficient coverage and minimal eddy-current distortions and pre-processed using MRTRIX (https://www.mrtrix.org/) and FSL (https://fsl.fmrib.ox.ac.uk/fsl/fslwiki/FDT/UserGuide). Diffusion tensor imaging parameters such as fractional anisotropy and mean diffusivity are derived using the *dtifit* function in FSL. We did not obtain a reverse-phase encoding scan, which precluded the use of tools like TOPUP (FSL) for correcting susceptibility-induced distortions. Please refer to Table 1, Table 2 and Fig. 1 for acquisition parameters of all scan sequences and details on small vessel disease quantification methods, respectively.

**Table 1:** MRI acquisition parameters in the PREVENT dementia programme

	TR (ms)	TE (ms)	Flip angle	Voxel size (mm^3^)	Slices	Duration (min:s)	Additional comments
T_1_-weighted	2300	2.98	9°	1.0 × 1.0 × 1.0	160	5:03	MPRAGE
T_2_-weighted	1500	80	150°	0.69 × 0.69 × 4.0	32	0:50	—
FLAIR	9000	94	150°	0.43 × 0.43 × 4.0	27	4:50	—
SWI	28	20	15°	0.72 × 0.72 × 1.2	72	5:05	—
ASL	2500	11	90°	3.0 × 3.0 × 3.0	14	4:20	PICORE; 50 averages
							Two variants differing in bolus duration (700 and 1675 ms)
DTI	11 700	90	90°	2.0 × 2.0 × 2.0	63	13:16	1 *b* = 0; 1000 s/mm^2^ volume, and 64 gradient directions
MRS	2000	30, 33 and 40	90°	20 × 20 × 20	Single voxel	3:52	PRESS with and without water suppression (96 and 16 averages, respectively)
BOLD fMRI	2000	30	80°	3.0 × 3.0 × 3.0	35	11:06	Resting state: 330 repetitions
						6:22	Task1: 188
						8:26	Task2: 250
Coronal hippocampal T_2_-weighted	6420	11	160°	0.41 × 0.41 × 2.0	20	6:20	—

ASL, arterial spin labelling; BOLD, blood oxygen level dependent; DTI, diffusion tensor imaging; fMRI, functional MRI; MRS, magnetic resonance spectroscopy; TR, repetition time; TE, echo time; FLAIR, fluid-attenuated inversion recovery; SWI, susceptibility-weighted imaging.

**Table 2:** Imaging markers of cerebral small vessel disease taken from Low *et al*.^20^ with permission (2022)

	White matter hyperintensities	Lacunes	Enlarged perivascular spaces	Cerebral microbleeds
What are they?	Patchy or diffuse lesions thought to represent axonal loss and demyelination	Focal subcortical infarcts caused by occlusion of perforating arteries	Microscopic fluid-filled spaces surrounding perforating vessels of the brain that become visible when dilated and also referred to as Virchow–Robin spaces	Small foci of chronic accumulation of blood products in brain tissue, Also referred to as microhaemorrhages
MRI sequence and appearance (+) Hyperintense/bright (−) Hypointense/dark	FLAIR (+)	T_1_-weighted (−) T_2_-weighted (+) FLAIR (−)	T_2_-weighted (+)	SWI (−)
Typical size	Variable	3–15 mm	<3 mm	2–5 up to 10 mm
Shape	Irregular Punctate/confluent	Round/ovoid	Axial view In centrum semi-ovale: rounded/linear In basal ganglia:round/ovoid, cyst-like	Round/ovoid
Method of quantification	Semi-automated quantification of volumes + Fazekas rating	Manual identification with cross-verification in T_1_, T_2_ and FLAIR scans	EPVS rating scale (range from 0–4)	Manual identification according to Microbleed Anatomical Rating Scale (MARS)

EPVS, enlarged perivascular spaces.

Resting-state blood oxygen level-dependent functional MRI of ∼10 min was acquired from each participant who was instructed to keep their eyes closed and not to think about anything specific. Participants also completed a task based (functional MRI), which was divided into two parts separated by ∼25–30 min. All participants had normal or corrected normal vision (MRI-compatible spectacles were available and supplied when necessary) and were provided with verbal instructions and an opportunity to practice responding before engaging in the task.

## Part 1 (∼6-min duration)

Participants were shown 37 indoor and 38 outdoor images (75 in total) randomly selected from a total of 50 indoor and 50 outdoor images. The images stayed on the centre of the screen for 3 s. The participant then had up to 2 s to respond by pressing one of the two buttons to indicate whether the image they saw was an indoor or outdoor scene. Participants were not informed they would be tested for their memory of these images at this stage.

## Part 2 (∼8-min duration)

After a delay of ∼25–30 min, participants were presented with 100 images (50 indoor scenes and 50 outdoor scenes) in pseudorandom order. Seventy-five of these images were already presented in the first part of the task, while 25 were new images. Each image was presented again for 3 s and participants had up to 2 s to indicate whether this is a previously seen or new image.

Analysis of data generated by the functional MRI task was conducted using SPM, RSA toolbox^21^ and in-house MATLAB scripts.

### Cognitive assessments

Participants completed a battery of cognitive assessments, in particular, focusing on cortical and sub-cortical brain regions hypothesized to be first affected in neurodegenerative disease, with a preference for early stages of Alzheimer’s disease. All experimental cognitive measures were selected by experts in the neuropsychology of ageing due to their ability to detect very subtle quantitative and qualitative changes in cognition.

#### The COGNITO

It is a computerized battery of tasks, designed to detect the widest possible range of cortical and sub-cortical deficits. The battery taking ∼45 min to complete includes the following sub-tests: reaction time; phonemic and syntactic comprehension; auditory and visual attention; visuospatial associative learning and working memory; immediate, delayed and cued visual and verbal recall; conceptual sequencing; naming; semantic access; and vocabulary.^22^ A tactile screen is used to capture response latencies and qualitative aspects of performance such as perseveration, proactive interference and visual field neglect.

#### The FMT

It is administered by a tablet device the Four Mountains Test (FMT) assesses the linkage between episodic and spatial functions of the hippocampus permitting representation of spatial information in an allocentric form and hence encoding of the context in which events occur.^23^ Computer-generated landscapes comprised of four hills (of varying shape and size) surrounded by a distant semi-circular mountain range are presented with a sample image for 10 s following which the subject is immediately presented with four alternative images. One of which (the target image) shows the same topography as the sample image, seen from a novel viewpoint, from which they must identify the target image by pressing a key. Non-spatial features (lighting, vegetation and weather conditions) of both target and foil landscapes are varied between presentation and testing, such that transient local features of the image cannot be relied on to solve the task. The task takes ∼15 min to complete.

#### The National Adult Reading Test

National Adult Reading Test is a 50-item word pronunciation test providing an indicator of premorbid intellectual functioning taking 10 min to complete.^24^

#### The VST

The Virtual Supermarket Trolley (VST) is sensitive to deterioration in the precuneus, retrosplenial cortex and entorhinal connections and measures egocentric spatial orientation (as opposed to allocentric) through the presentation of 14 video vignettes in an ecological virtual supermarket from a first-person perspective.^25^ A route is taken through a supermarket in which the participant is behind the trolley and involves a series of 90° turns and at the end the subject is required to point in the direction of the entry. The task is also administered through a computerized tablet device, but responses are recorded on paper by a researcher.

#### The Visual Short-Term Memory Binding Test

Visual Short-Term Memory Binding Test assesses memory binding abilities using combinations of shapes and colours on a computerized assessment taking ∼15 min to complete. The test has been shown to predict familial Alzheimer’s disease 10–15 years prior to the onset of clinical symptoms and is therefore a critical test to be used in this group.^26^

#### Addenbrookes Cognitive Examination 3

The ACE-III provides a brief screen of possible memory, attention, fluency, language and visuospatial disabilities. The test was included following the pilot data collection to include a clinically validated measure of cognition to ensure there were no pre-existing signs of cognitive impairment, which would exclude participants from the study.^27^ The test is a pen-and-paper assessment, taking ∼15 min to complete.

### Self-report questionnaires

Participants completed a series of self-report questionnaires covering multiple lifestyle and risk factor domains. These included questionnaires on pregnancy and menstruation, the Lifetime of Experiences Questionnaire,^28^ history of educational attainment, physical activity,^29^ musical expertise, depression (Center for Epidemiologic Studies—Depression Scale),^30^ anxiety (State-Trait Anxiety Inventory),^31^ sleep (Pittsburgh Sleep Quality Index),^32,33^ resilience (Connor-Davidson Resilience Scale),^34^ stressful life events (Life Stressor Checklist-Revised),^35^ traumatic brain injury (Brain Injury Screening Questionnaire)^36^ and diet (Scottish Collaborative Group Food Frequency Questionnaire).^37^

### Sub-studies

Alongside the main PREVENT study, various researchers from institutions across the UK, Ireland and France have joined as collaborators to recruit PREVENT participants to additional sub-studies (Table 3). Data from these studies will be added to the main PREVENT database following embargo periods.

**Table 3:** Overview of sub-studies which have recruited PREVENT participants

Study name	Lead researcher	Study description
Linguistic markers of future risk for Alzheimer’s disease	Professor Alison Wray, University of Cardiff	Online assessment where language use was analysed to investigate whether linguistic markers could identify potential risk for future Alzheimer’s disease. A total, of 179 participants completed the baseline assessment, and 35 were followed up 2 years later to identify any changes in language use across this time period.
Approaches to the Communication of Alzheimer’s disease risk (ACAR study)	Dr Richard Milne, University of Cambridge	Focus groups held with research participants investigating attitudes to communication of future risk of dementia. Sixteen PREVENT participants were recruited alongside additional volunteers recruited from other research studies across Europe. The focus groups were structured to explore participants’ interest in learning about their Alzheimer’s disease risk and what their preferences were around disclosure.^38^
PREVENT-Elicitation of Dialogues (PREVENT-ED) study	Dr Sofia De La Fuente Garcia	Collected speech data from 43 participants enrolled at the Edinburgh site while they engaged in a cognitively stimulating task. Audio recordings were processed for speech features and machine learning methods used to test for associations between these features and risk factors collected in the PREVENT cohort.^39^
Mobile-technologies for the Assessment of Cognition (MTAC) study	Dr Ivan Koychev, University of Oxford	Thirty-five participants recruited from PREVENT Oxford site. Explored usability of a smartphone-based application to track cognition and function and positional technology using interactions between a smartwatch and Bluetooth beacons positioned around the homes to assess the level of function, activity and ability to navigate the environment.
*Neureka*	Dr Claire Gillan, Trinity College Dublin	Ninety-four PREVENT participants completed cognitive assessments via a mobile phone-based application to assess the validity of these tests in comparison with gold standard in person assessments.
Oral Health in PREVENT	Prof Angus Walls, University of Edinburgh	Pilot study was conducted at the Edinburgh and Dublin PREVENT sites investigating periodontal disease and future risk for dementia. Participants were invited for a dental examination including a dental X-ray and provided plaque and saliva samples.
Sunrise in PREVENT study	Prof Yves Dauvilliers, University of Montpellier	Participants were invited to wear a sleep activity recording device attached to their chin, which measures mandibular movements to monitor sleep behaviour and aid the diagnosis of obstructive sleep apnoea.
Barriers, facilitators and motivators to dementia prevention research	Dr Laura Booi, Leeds Beckett University	Aimed to explore what facilitates participation in dementia prevention research and what might be barriers. Recruitment was targeted to those from seldom heard groups in research to try and understand what challenges there might be to research participation. Interviews were conducted with 19 participants and analysed using thematic analysis.
Fear about memory loss in midlife	Dr Francesca Farina, Northwestern University, USA	Participants completed an online assessment exploring fear of memory loss in midlife. Various scales related to fear and avoidance were conducted by participants to understand the level of fear certain individuals may have about dementia while in midlife and whether this may impact their social behaviour.
Auditory Attention in Cognitive disorder (AudCog)	Dr Meher Lad, Newcastle University	Participants were invited to complete an online auditory assessment using multiple tasks to assess auditory function (pure-tone audiometry, speech-in noise perception task, auditory figure ground task, auditory working memory task and auditory sequence learning task).

### Retinal imaging in PREVENT

Participants at the Edinburgh site are invited to undergo a retinal imaging protocol. Imaging the retina is a non-invasive and relatively easy process, making it an ideal area to investigate for translation to clinical practice. Evidence is accumulating that implicates microvasculature in neurodegenerative disease aetiology,^40-43^ with drusen on the retina more prevalent in Alzheimer’s disease.^44^ The retinal imaging sub-study aims to investigate retinal imaging measures in relation to dementia risk in PREVENT.

### Amyloid imaging in PREVENT study

Up to 200 PREVENT participants are being invited to take part in the Amyloid Imaging in Prevent study. This study involves undergoing a PET-CT scan to measure amyloid deposition in the brain. The tracer 18F-florbetaben is used for this study, and participants are scanned on a single-site scanner located in London.

### Tau imaging

A sub-group of 31 PREVENT participants from the Amyloid Imaging in PREVENT study also participated in a tau imaging study, to additionally measure levels of tau in their brain using PET imaging. For this study, the 18F-PI-2620 tracer was used, and the participants were scanned on a single scanner at the Imanova Centre for Imaging Sciences in London.

### The ENtorhinal CoRtex structure and function in PREVENT study

The aim of the ENtorhinal CoRtex structure and function in PREVENT study is to investigate whether the structure and function of the entorhinal cortex may be impaired in midlife in those who may be at a higher risk of future Alzheimer’s disease. One hundred participants completed virtual reality tasks, with a subset of 55 participants additionally completed a 7-T MRI brain scan. Scans were structural images with a high-resolution entorhinal–hippocampal circuit field-of-view and functional images aiming to measure entorhinal grid cell-like activity from the anterior-medial entorhinal cortex subdivision.

The virtual reality task was a test of path integration, a behaviour thought dependent on specialized grid cell spatial cell populations found in the anterior-medial entorhinal cortex. Virtual Reality testing was required to enable full participant self-motion and limit access to proximal visual cues, thought critical for engaging entorhinal cortex during spatial navigation.

### Football and rugby cohort

In addition to the main cohort, the PREVENT programme is being further developed through the recruitment of participants who are ex-professional football (Brain Health Outcomes in former Professional and Elite athletes) or rugby (PREVENT-Rugby Footballer Cohort) players. In total, 210 ex-professional or elite players (male and female) will be recruited allowing for focused analyses exploring specific early indicators of disease for players from these sports and comparing to non-sports players from the wider PREVENT cohort.

### Operational organization

#### Steering committee

The PREVENT programme is managed by a steering group committee comprising of the principal investigators from each site, two representatives from the PREVENT participants’ panel (detailed below), the study statistician and other academics from relevant disciplines with a key role in managing the research programme. Meetings are held quarterly to discuss study progress, funding plans, study developments, such as any new sub-studies in the pipeline, and any other core study business. This steering group committee also reviews and approves data and sample access requests and project proposals.

#### Participant and public involvement panels

Participant and public involvement has been at the core of the PREVENT dementia programme since its inception, with the establishment of a participant panel during the study design phase. The original participant panel was set up to support the London centre of the project through the pilot phase, and two members of the panel were elected to sit on the steering committee. As the project has expanded to multiple centres and countries, the original panel have moved to support the wider project. The participant panel set-up is well described elsewhere.^45^ Briefly, the core panel consists of seven participants and one non-participant who meet with the chief investigator (C.W.R.) and national coordinator (K.W.) quarterly. The aim of the panel meetings is to discuss project progress, future aims, sub-studies and proposed analyses. To date, the panel has had a significant and positive impact on the project, supporting with recruitment, inclusion of additional sub-studies, understanding the participant experience and contributing to the future of the study.^45^ In addition to this, a participant panel has been established at the Edinburgh site, to support with the large number of sub-studies active at that centre. The Edinburgh panel was established in 2019 via advertisements to all active participants and has met once in person and multiple times online. The panel has supported reviews of sub-studies and supported staff to make decisions about approaches for recruitment to aforementioned sub-studies. The panels also help to co-develop any events aimed at participants such as annual conferences to share study findings.

#### Data management and quality control

As a first quality control step, study monitoring is carried out on a regular basis by the national coordinator as delegated by the sponsor and Principal Investigator (PI); study documentation is reviewed for errors and omissions at all study sites. The data are entered electronically onto the REDCap data management system.^46^ REDCap is a web-based software platform designed to support research data capture and management, hosted at the University of Edinburgh and managed by the study team. The system generates queries for research staff at the point of data entry. The creation of the project into REDCap replicates the same structure as the Case Report Form. This ensures that all the information from the Case Report Form is captured and stored properly when it is entered electronically. The design of the project into REDCap includes several countermeasures to ensure the best possible quality of the extracted data. Within REDCap, fields that contain important values are designed to be mandatory to store the necessary data, with flags alerting users to any omissions. Field restrictions have been implemented to avoid mistakes and prevent data entries from being inaccurate. For example, dates are checked for values that are outside of specific ranges, as well as being in the expected form, such as integer, date, time and text. Branching logic ensures certain fields remain hidden to research staff if the participant was not eligible to answer specific questions, avoiding the possibility of entering data in inaccurate fields.

Raw imaging data are transferred and backed up in the University of Cambridge XNAT platform. The unprocessed MRI data along with derived imaging maps and quantified neuroimaging measures are reviewed on a case-by-case basis by the PREVENT dementia imaging team in Cambridge. Visual assessments along with derived quality measures capturing signal and contrast to noise ratio are employed to assess image quality, where appropriate (e.g. imaging artefacts) scans or derived measures are excluded from further analysis. All scans were reviewed at each site and any incidental findings were reported back to the study team, who then fed back to the participants, and where relevant, their primary care practitioners.

Patient identifiable information (e.g. name and date of birth) was removed from the raw MRI DICOM data for every participant in each site using available protocols. This information was removed prior to sharing the scans with the central PREVENT dementia XNAT imaging database in Cambridge. MR images to be shared via the Dementia Platforms UK platform will further be defaced using software tools such as MRI reface.^47,48^ Data integration and pseudonymization protocols consistent with data protection principles outlined in the UK General Data Protection Regulation are finally applied prior to data release for research.

#### Data access

Open data access is an underpinning principle of the PREVENT dementia programme, with ambitions that data collected through this study will be critical to understanding brain health in the midlife period. The data set has already been highly requested and resulted in several publications from outside the core study team (see Table 4). The addition of the data to the ADDI platform is anticipated to increase the accessibility and use of this novel data set especially to low and middle-income countries.

**Table 4:** Data and sample access requests from study inception to July 2023 as well as publications arising from the PREVENT cohort from study inception to July 2023

Data requests and publications	Number
Data access requests	
Internal to consortium	62
External to consortium	44
Sample access requests	
Internal to consortium	3
External to consortium	3
Publications relating to PREVENT as of 1 July 2023	33 (18 imaging, 7 cognition, 8 other topics); see https://preventdementia.co.uk/publications/.

#### Descriptive statistics

Descriptive statistics are presented in the results section to provide an overview of the cohort, profiled by key demographics, cognitive health, key MRI measures and prevalence of known risk factors for dementia. Descriptive demographic data are presented for the full cohort. Cognitive data and prevalence of risk factors are presented for the full cohort, by risk group and by sex. Risk groups were determined *a priori* prior to data collection as follows: low risk for future dementia, no reported parental history of dementia and not an *APOEɛ4* carrier; medium risk for future dementia, either a parental history of dementia or an *APOEɛ4* carrier; and high risk for future dementia, both a parental history of dementia and an *APOEɛ4* carrier. Data are presented by risk groups and sex to provide a breakdown of the key variables by groups that may be of interest for future hypothesis-driven analyses. Descriptive statistics are provided for key MRI parameters with linear regression models used to detail associations with sex and age for comparison with other data sets.

## Results: description of PREVENT v700.0 data set

### Demographics and APOEɛ4 descriptive statistics

The baseline data set includes 700 participants, with the majority of participants recruited at the Edinburgh (*n* = 222, 31.7%) and London sites (*n* = 210, 30.0%) (Table 5).

**Table 5:** Number of participants in final data set from each site

Site	*N* (%)
Cambridge	100 (14.3)
Dublin	100 (14.3)
Edinburgh	222 (31.7)
Oxford	68 (9.7)
London	210 (30.0)

There is a predominance of female participants (*n* = 433, 61.9%) at all sites except Dublin (Supplementary Fig. 1), with a nearly even split on those with and without parental history of dementia (has parental history, *n* = 360, 51.4%) resulting from the targeted recruitment method used. Participants had a mean age of 51.17 years (±5.47) at baseline, were highly educated (mean: 16.69 ± 3.44 years) and had high prevalence of *APOEɛ4* carriers [*n* = 264/694 (38.0%) of which 34 (4.9%) homozygotes]. There were no differences in *APOEɛ4* by site (Supplementary Fig. 1). The cohort mainly included participants of European Ancestry (*n* = 672, 95.96%). Participants are categorized into high (positive parental history and *APOEɛ4* carrier), medium (either positive family history or *APOEɛ4* carrier) and low (neither family history nor *APOEɛ4* carrier) risk groups, with an approximately even split across the three risk groups (high risk for future dementia: 232, 33.4%; medium risk for future dementia: 305, 43.9%; low risk for future dementia: 157, 22.6%). Full descriptive details are available in Table 6.

**Table 6:** Demographics of total cohort

Variable	Mean (SD)/*N* (%)	
Education (years)	16.69 (±3.44)	
	Range: 0–38 years	
Parental history of dementia	360 (51.4%)	
Sex (female)	433 (61.9%)	
Age (years)	51.17 (±5.47)	
	Range: 40–60^a^	
	No family history	With family history
*APOEɛ4* non-carrier	Low risk for future dementia	Medium risk for future dementia
	*N* = 232, 33.4%	*N* = 198, 28.5%
*APOEɛ4* carrier	Medium risk for future dementia	High risk for future dementia
	*N* = 107, 15.5%	*N* = 157, 22.6%
Estimated years until dementia onset (*n* = 348)^b^	23.07 (±7.27) years	

^a^Two participants were aged 60 at the time of baseline demographic data collection, data excluded from descriptive statistics presented in Table 6.

^b^Sample size is determined by the number of people with a reported parental history of dementia (*n* = 360) who provided an age of onset for their parent’s dementia. Where both parents were reported to have a diagnosis of dementia, the age of the youngest onset was used.

### Cognitive domains overview

Cognitive impairment was screened for by the ACE-III (note results not available for *n* = 233 participants at baseline as incorporated via a protocol amendment after these visits were complete). Mean cognitive scores for the cohort and by risk group and sex are presented for the ACE-III, COGNITO tasks, FMT and VST in Table 7. A full breakdown of all COGNITO scores is presented in Supplementary Tables 1 and 2. Linear regression models were used to explore significant associations between cognitive scores and either risk group or sex.

**Table 7:** Cognitive scores in total cohort by risk group and by sex

Domain	Cognitive test	Total [mean (SD)]	Low risk [mean (SD)]	Medium risk [mean (SD)]	High risk [mean (SD)]	Female [mean (SD)]	Male [mean (SD)]
General cognition	ACE-III^a^	95.57 (4.06)	95.95 (4.29)	94.99 (4.24)	96.12 (3.19)	96.17 (3.87)	94.73 (4.17)
		Range 66–100					
Attention	Visual and auditory attention^b^	9.82 (0.45)	9.82 (0.48)	9.82 (0.47)	9.85 (0.37)	9.78 (0.51)	9.90 (0.33)
		Range 7–10					
Memory	Face-Name Recognition^c^	5.26 (2.11)	5.20 (2.20)	5.19 (2.15)	5.50 (1.94)	5.69 (2.02)	4.57 (2.08)
		Range 0–9					
	Implicit Memory^c^	1.03 (0.67)	1.02 (0.64)	1.04 (0.67)	1.00 (0.73)	1.02 (0.69)	1.04 (0.65)
		Range −2.6–5.6					
Visuospatial abilities	Geometric Forms^c^	6.36 (1.17)	6.35 (1.11)	6.29 (1.20)	6.52 (1.18)	6.37 (1.20)	6.35 (1.11)
		Range 1–8					
Language	Phoneme Comprehension^c^	8.62 (0.58)	8.69 (0.57)	8.61 (0.60)	8.55 (0.58)	8.65 (0.57)	8.58 (0.60)
		Range 6–9					
	Verbal Fluency (semantic)^d^	16.36 (4.16)	15.99 (3.94)	16.56 (4.33)	16.54 (4.16)	17.44 (3.97)	14.68 (3.88)
		Range 0–29					
	Verbal Fluency (phonemic)^c^	11.26 (4.10)	11.21 (4.05)	11.13 (4.14)	11.60 (4.14)	11.42 (4.11)	11.00 (4.08)
		Range 1–24					
Egocentric spatial orientation	VST^e^	10.48 (2.19)	10.62 (2.25)	10.48 (2.17)	10.37 (2.19)	10.12 (2.39)	10.97 (1.79)
		Range 1–12					
Allocentric spatial orientation	FMT^f^	10.36 (2.41)	10.31 (2.34)	10.39 (2.50)	10.34 (2.41)	10.28 (2.38)	10.46 (2.46)
		Range 0–15					

^a^Total: *n* = 467; low risk: *n* = 151; medium risk: *n* = 205; high risk: *n* = 105; female: *n* = 267; male: *n* = 197.

^b^Total: *n* = 691; low risk: *n* = 228; medium risk: *n* = 301; high risk: *n* = 156; female: *n* = 428; male: *n* = 263.

^c^Total: *n* = 693; low risk: *n* = 228; medium risk: *n* = 302; high risk: *n* = 157; female: *n* = 428; male: *n* = 265.

^d^Total: *n* = 692; low risk: *n* = 228; medium risk: *n* = 302; high risk: *n* = 156; female: *n* = 427; male: *n* = 265.

^e^Total: *n* = 453; low risk: *n* = 141; medium risk: *n* = 202; high risk: *n* = 105; female: *n* = 260; male: *n* = 193.

^f^Total: *n* = 459; low risk: *n* = 147; medium risk: *n* = 201l; high risk: *n* = 105; female: *n* = 262; male: *n* = 197. ACE-III, Addenbrookes Cognitive Examination III; FMT, Four Mountains Test; VST, Virtual Supermarket Trolley.

### COGNITO, FMT and VST

Mean scores for key COGNITO tasks, the FMT and VST are presented in Table 7 for the full cohort, by the three risk groups for future dementia and by sex. There are no normative values for these cognitive tasks, and as such, the ranges of scores from participants in PREVENT are provided in the table to provide context to the mean and standard deviations. Scores on COGNITO tasks were generally comparable across the three risk groups for future dementia. Some apparent sex differences emerged across the COGNITO tasks, with female participants performing better on a memory task of face and name recognition [female: 5.69 (±2.02); male: 4.57 (±2.08)] and a language task of semantic verbal fluency compared with male participants [female: 17.44 (±3.97); male: 14.68 (±3.88)].

### Addenbrookes Cognitive Examination 3

Data from the ACE-III are available for 464 participants at the baseline visit; this assessment was added part way through the baseline data collection; hence, data are not available for all participants at baseline. When applying a clinical cut-off of 88 (recommended dementia caseness cut-off for sensitivity^49^), there are 30 participants: seven in the low-risk group (age range 40–59, 42.9% female); 20 in the medium-risk group (age range 41–59, 40% female) and three in the high-risk group (age range 49–55, all male). When using a clinical cut-off of 82 (recommended dementia caseness cut-off for specificity^49^), two participants score at or below this (one in the low-risk group and one in the medium-risk group).

### Imaging overview

From the completed 666 scans, 17 were excluded from analyses due to incidental findings (e.g. meningiomas) or poor quality of the imaging data. Our sample had an average WMH volume of 2.26 ± 2.77 ml (*n* = 643; median = 1.39 ml). This was higher than the mean of 0.95 ml in another midlife cohort of participants with a mean age of 45.^50^ However, this was expected given that our sample was older (mean age 51.2 years) and enriched for family history of dementia, which may explain the high prevalence of *APOEɛ4* carriers (37.7%) compared with the expected population prevalence of 20%. A small proportion (6.2%; *n* = 40 out of 647) had a high burden of WMH, as defined by a Fazekas score of 3 in the periventricular area or a score of 2 or more in the deep subcortical white matter.^51^ WMH volume did not differ by *APOEɛ4* status or family history of dementia in unadjusted analysis or adjusted analyses controlling for sex, age, education and site.^18^ WMH burden increased with older age in both the unadjusted (*t* = 8.15, *P* < 0.001) and adjusted analysis controlling for sex, education and site (*t* = 7.91, *P* < 0.001). Males (2.99 ml) had greater WMH volumes than females (1.81 ml), even after normalizing for head size [(WMH volume/intracranial volume) ∗ 100%; males = 0.16, females = 0.11]—results were significant in both unadjusted (*ρ* = 0.25, *P* < 0.001) and covariate-adjusted analyses of WMH burden (*t* = 5.41, *P* < 0.001).

Following analysis with the FreeSurfer software (version 7.1.0), 623 data sets were free of incidental findings and artefacts and with good quality data following the implementation of the *recon-all* pipeline. The mean hippocampal volume for the cohort (left and right hemispheres) was 8.15 ± 0.79 ml with an estimated total intracranial volume of 1490.6 ± 163.1 ml, grey matter volume of 646.3 ± 58.0 ml and cerebral white matter volume of 466.5 ± 56.8 ml. Mean cortical thickness was 2.43 ± 0.07 mm. In linear regression analysis with age, sex, education years, study site, estimated total intracranial volume and *APOEɛ4* as predictors of hippocampal volume, sex was a significant predictor with females having smaller volumes (*t*_female_ = −3.03, *P* < 0.01). In a similar model predicting total GM volume, age (*t*_age_ = −4.84, *P* < 0.01), sex (*t*_female_ = −10.44, *P* < 0.01) and education years (*t*_educ_ = 2.24, *P* = 0.03) were all significant predictors. Finally, mean cortical thickness was predicted by age (*t*_age_ = −4.73, *P* < 0.01) and years of education (*t*_educ_ = 2.19, *P* = 0.03).^19^ Further description of the cohort imaging findings will be presented in an upcoming manuscript.

### Prevalence of risk factors for Alzheimer’s disease

In Table 8, we report the prevalence of common risk factors for Alzheimer’s disease as defined by the 2020 Lancet Commission on dementia prevention^52^ as well as sleep as an important risk factor for brain health.

**Table 8:** Prevalence of modifiable risk factors for dementia in total cohort and by risk group and by sex

Life stage	Risk factor	Total cohort	Low risk for future dementia	Medium risk for future dementia	High risk for future dementia	Female	Male
Early life	Education <13 years	11.4%	10.3%	11.8%	12.1%	11.1%	11.9%
Midlife	Hearing loss	11%	11.2%	14.6%	10.8%	8%	13.9%
	Head injury (blow to head with loss of consciousness)	35.6%	37.5%	36.9%	31.6%	27.4%	49.6%
	Stage II hypertension	16.7%	16.4%	17.0%	16.6%	9.7%	28.1%
	Anti-hypertensive medication	7.7%	7.8%	8.2%	6.4%	5.3%	11.6%
	Alcohol units >14 units/week	24.8%	25.0%	26.0%	23.2%	18.4%	35.4%
	Obesity	27.1%	28.0%	25.9%	28.7%	26.3%	28.5%
Later life	Smoking (current)	5.6%	5.2%	7.5%	2.5%	4.6%	7.1%
	Depression (CES-D ≥ 16)	16.7%	20.7%	13.4%	17.2%	16.6%	16.9%
	Anti-depressant medication	8.0%	6.5%	8.9%	7.6%	8.3%	7.5%
	Social isolation (social interaction less than once a week)	8.5%	12.1%	8.3%	3.1%	7.9%	9.7%
	Physical inactivity	47.4%	48.5%	48.2%	46.5%	53.4%	38.6%
	Diabetes	3.3%	3.9%	3.0%	3.2%	2.8%	4.1%
	Poor sleep (Buysse scoring methodology and cut off criteria^32^)	45.0%	47.8%	43.3%	47.8%	47.1%	41.6%

CES-D, Center for Epidemiologic Studies—Depression Scale.

## Discussion

The PREVENT dementia programme is a multi-site study with a comprehensive and deeply phenotyped baseline data set from 700 participants recruited in midlife, an estimated 23 years from estimated dementia onset based on parental age of dementia onset. Data are available across a number of key early neurodegenerative disease indicators and risk factors for future neurodegenerative disease. Importantly, this data collection has been collaboratively designed with an engaged participant panel. PREVENT participants are generally young and cognitively healthy. However, of importance to the field of dementia prevention, risk factors are already beginning to accumulate in this group. Of note, three-quarters (76.6%) of the cohort reported at least one head injury, 64.5% were overweight or obese, 47.4% were physically inactive, and 45% had poor sleep. Male participants were carrying more of this burden, with higher rates of hearing loss, hypertension, being overweight, current smoking, TBI, alcohol use and diabetes. This midlife accumulation of risk factors highlights the importance of studying the origins of neurodegenerative disease in this age group. In fact, emerging evidence suggests that risk factors confer differential effects on brain health across the lifespan, whereby various risk factors are more predictive when measured at midlife, relative to late life.^53-56^

Given the early age and minimal cerebrovascular burden in the PREVENT cohort, it is well suited to delineate some of the earliest changes associated with risk factors of *APOEɛ4* and family history while mitigating risks of confounds from co-morbidity. The cognitive data presented in the manuscript showed no significant difference by *a priori* risk groupings but did suggest a number of sex differences in cognitive performance. As this manuscript was designed to be descriptive rather than hypothesis driven, the analysis was not designed to test any hypothesis regarding sex differences in midlife cognition; however, the findings suggest that further research in this topic is warranted, particularly given the emerging evidence in sex differences in the accumulation of Alzheimer’s disease pathology.^57^

The collaborative core of PREVENT with both established cohorts (such as ALFA) and onboarding new sub-studies allows for both replication efforts and enrichment of the cohort. In particular, some of the sub-studies will provide data to support profiling of PREVENT participants using the Amyloid–Tau–Neurodegeneration criteria as well as analysis of stored blood using recently developed assays. These developments will allow researchers to study interactions between these pathological Alzheimer’s disease hallmarks with *APOEɛ4* and family history of dementia. There is also an opportunity to address questions that have not received much attention in the literature to date. For example, can data from the PREVENT cohort help us to understand whether parental subtype of dementia is consequential and whether Alzheimer’s disease-type parental dementia is associated with more deleterious outcomes versus non-Alzheimer’s disease parental dementia?

There are some notable limitations to the PREVENT cohort, namely around representative diversity. Particularly, there is a lack of diversity in the ethnicity of participants, with the majority identifying as Caucasian, which has implications for both genetic analysis and the generalizability of findings to the UK and global populations. The cohort is also comparatively higher educated than the general adult population in the UK, which may limit the generalizability of results to all groups of society.

The true potential of PREVENT is likely to be realized through both the release of the baseline data to the wider scientific community through the ADDI platform and continued data collection. Additional and ongoing longer-term follow-ups will also be beneficial to explore the symptomatic consequences of early pathological disease accumulation.

## Supplementary Material

fcae189_Supplementary_Data

## Data Availability

The baseline data set is available to access through a data request on the study website (www.preventdementia.co.uk); the ADDI platform baseline data set DOI: https://doi.org/10.34688/PREVENTMAIN_BASELINE_700V1; Dementia Platforms UK; and the Global Alzheimer’s Association Network. For imaging data, a number of derived variables (for example the global volumetrics and WMH volume) are available in the ADDI data set. Raw, defaced imaging data will be made available in the future to access upon request. Code used to generate summary statistics is available on the following link: Baseline 700 data code.
