# Supplementary material for: The PREVENT dementia programme: baseline demographic, lifestyle, imaging and cognitive data from a midlife cohort study investigating risk factors for dementia
Source: Brain Commun. 2024 May 31;6(3):fcae189. doi: 10.1093/braincomms/fcae189 (PMC11166176; doi:10.1093/braincomms/fcae189)
Supplement: fcae189_Supplementary_Data [file fcae189_supplementary_data.docx]

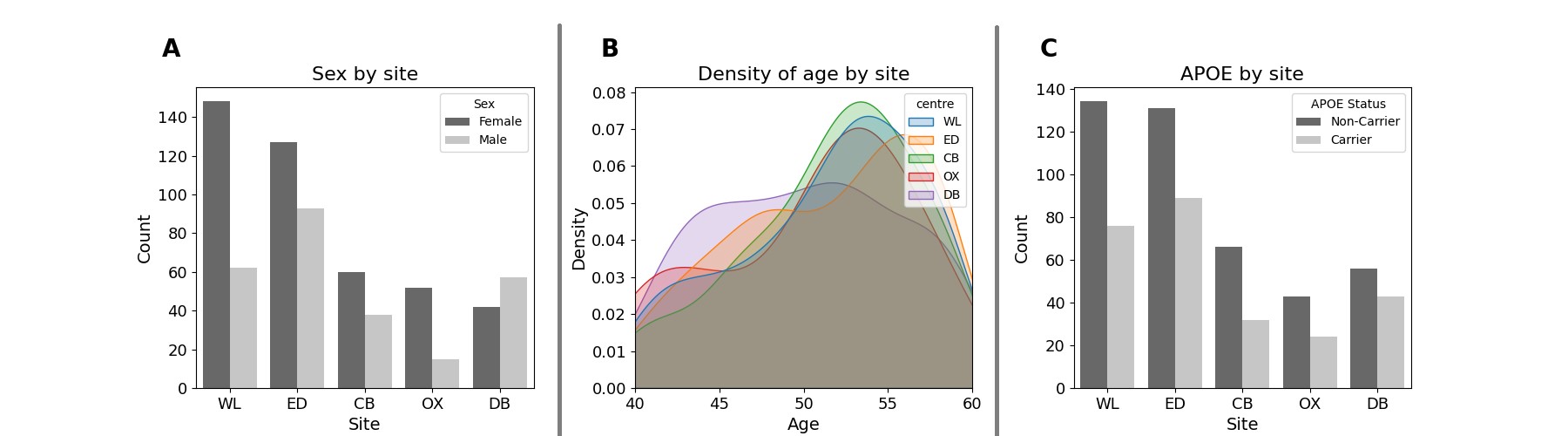


**Supplementary Figure 1:** Distribution of sex (A), age (B) and *APOEɛ4* (C) by site (CB: Cambridge, DB: Dublin, ED: Edinburgh, OX: Oxford; WL: London).

|  |  | **Sample**  **Size** | **PCT_1%** | **PCT_5%** | **PCT_10%** | **PCT_25% Q1** | **PCT_50% Median** | **PCT_75% Q3** | **PCT_90%** | **PCT_95%** | **PCT_99%** |
| --- | --- | --- | --- | --- | --- | --- | --- | --- | --- | --- | --- |
| EP01M | Task 1: reaction time, mean (millisec) | 695 | 262 | 277 | 287 | 306 | 328 | 359 | 388 | 410 | 455 |
| EP03BR | Task 3: sentence comprehension, No correct answers | 700 | 3 | 4 | 4 | 5 | 5 | 5 | 5 | 5 | 5 |
| EP04BRT | Task 4: Auditory attention, total number of correct counts (max=10) | 699 | 7 | 8 | 9 | 10 | 10 | 10 | 10 | 10 | 10 |
| EP05BRT | Task 5: Total correct answers (max=10=5 trials with 2 forms) | 700 | 7 | 9 | 9 | 10 | 10 | 10 | 10 | 10 | 10 |
| EP05T1M | Task 5: Auditory attention, mean time (millisec) until 1st click (whatever the response) | 700 | 1687 | 2067 | 2248.5 | 2647.5 | 3259.5 | 3994.5 | 4909 | 5541 | 7363.5 |
| EP06BRT | Task 6: Total number of correct answers for form recognition (max=10=5*2) | 698 | 8 | 9 | 9 | 10 | 10 | 10 | 10 | 10 | 10 |
| EP06T1M | Task 6: Working memory, mean time (millisec) until 1st click (whatever the response) | 698 | 1623 | 1948 | 2130 | 2513 | 3113 | 4022 | 4973 | 6233 | 8907 |
| EP07ET02YT | Task 7, level 2: Mean duration of answers (milliseconds) | 696 | 686 | 749 | 791 | 856 | 941 | 1029 | 1143 | 1213 | 1423 |
| EP07ET03BR | Task 7, level 3: Stroop proactive inference, number of correct answers | 696 | 4 | 13 | 15 | 17 | 18 | 20 | 21 | 22 | 23 |
| EP08BR | Task 8: Immediate name recall, number of names correctly recalled | 699 | 4 | 4 | 5 | 6 | 7 | 8 | 8 | 9 | 9 |
| EP10RE | Task 10: Spatial span, number of successes | 699 | 0 | 2 | 3 | 3 | 5 | 5 | 6 | 6 | 6 |
| EP10T | Task 10: Spatial span, mean time (milliseconds) | 699 | 880 | 1303 | 1420 | 1764 | 2234.2 | 2918.5 | 3605.8 | 4283.5 | 5570.7 |
| EP11BR | Task 11, geometric forms: No correct responses | 700 | 3 | 4 | 5 | 6 | 7 | 7 | 8 | 8 | 8 |
| EP11MY | Task 11, Mean time for correct responses (milliseconds) | 700 | 3101.5 | 3629.5 | 3973.5 | 4740 | 5592.5 | 6608.5 | 7839 | 8663 | 9871.5 |
| EP12BR | Task 12: Phoneme comprehension, No of correct answers (max=10) | 700 | 7 | 7 | 8 | 8 | 9 | 9 | 9 | 9 | 9 |
| EP12MY | Task 12, word comprehension: mean time for correct response (milliseconds) | 700 | 943 | 1097.5 | 1151.5 | 1273 | 1420 | 1617 | 1834.5 | 1955 | 2272 |
| EP13BA_P | Task 13: Semantic associations, No correct associations(max=10) | 700 | 8 | 9 | 9 | 9 | 10 | 10 | 10 | 10 | 10 |
| EP13RDP | Task 13: Semantic associations, No trials over time limit | 700 | 0 | 0 | 0 | 0 | 0 | 0 | 0 | 0 | 1 |
| EP13RI | Task 13: Semantic associations, No of exact responses for naming | 700 | 8 | 9 | 10 | 10 | 10 | 10 | 10 | 10 | 10 |
| EP14BR_P | Task 14: Visuospatial logic (matrices), nb of correct answers | 699 | 3 | 6 | 7 | 8 | 9 | 11 | 12 | 13 | 14 |
| EP14TT | Task 14: Visuospatial logic (matrices), Total time | 699 | 84387 | 104287 | 114612 | 139216 | 177955 | 229689 | 278682 | 309734 | 371314 |
| EP17ET01BR | Task 17, Delayed name recall, free recall: no of correct answers | 699 | 3 | 4 | 5 | 6 | 7 | 8 | 9 | 9 | 9 |
| EP17ET02BR | Task 17, Delayed name recall, cued recall: no of correct answers | 700 | 3 | 4 | 5 | 6 | 7 | 8 | 9 | 9 | 9 |
| EP18BN | Task 18, surname recognition: no. of names correctly recognized | 700 | 1 | 2 | 2 | 4 | 5 | 7 | 8 | 9 | 9 |
| EP19_20C | Tasks 19 and 20, verbal fluency, semantic phonemic cue: nb of correct answers 60s | 699 | 14 | 17 | 19 | 23 | 28 | 32 | 37 | 39 | 43 |
| EP21T | Task 21, narrative story: total nb of correct answers (max=27) | 700 | 3 | 7 | 8 | 11 | 14 | 17 | 20 | 21 | 24 |
| EP22T | Task 22, descriptive story: total nb of correct answers (max=27) | 700 | 5 | 6.5 | 8 | 10 | 13 | 17 | 19.5 | 21 | 23.5 |
| EP23BR | Task 23: Nb of correct answers (max=35) | 700 | 8 | 12 | 14 | 18 | 21 | 25 | 28 | 29 | 32 |
| EP23MR | Task 23: Vocabulary, no of incorrect answers | 700 | 3 | 6 | 7 | 10 | 13 | 17 | 21 | 23 | 26 |
| EP24DF | Task 24 implicit memory: difference | 700 | -0.4 | 0 | 0.2 | 0.6 | 1 | 1.4 | 1.8 | 2.1 | 2.6 |
| EP25T_P | Task 25: scoring the design | 607 | 45 | 55 | 62 | 68 | 73 | 77 | 80 | 80 | 80 |

**Supplementary Table 1:** Percentiles of COGNITO tasks in all participants.

|  |  | Sex | Education | Sample Size | PCT_1% | PCT_5% | PCT_10% | PCT_25% Q1 | PCT_50% Median | PCT_75% Q3 | PCT_90% | PCT_95% | PCT_99% |
| --- | --- | --- | --- | --- | --- | --- | --- | --- | --- | --- | --- | --- | --- |
| EP01M | Task 1: reaction time, mean (millsec) | Male | <18 years | 175 | 262 | 276 | 284 | 303 | 322 | 348 | 369 | 394 | 431 |
|  |  | Male | 18+ | 90 | 253 | 273 | 285.5 | 300 | 319.5 | 352 | 390 | 412 | 460 |
|  |  | Female | <18 years | 255 | 262 | 280 | 297 | 315 | 342 | 374 | 398 | 425 | 444 |
|  |  | Female | 18+ | 175 | 264 | 278 | 287 | 302 | 323 | 352 | 384 | 408 | 456 |
| EP03BR | Task 3: sentence comprehension, No correct answers | Male | <18 years | 176 | 3 | 4 | 4 | 5 | 5 | 5 | 5 | 5 | 5 |
|  |  | Male | 18+ | 91 | 3 | 4 | 4 | 5 | 5 | 5 | 5 | 5 | 5 |
|  |  | Female | <18 years | 256 | 2 | 3 | 4 | 5 | 5 | 5 | 5 | 5 | 5 |
|  |  | Female | 18+ | 177 | 3 | 4 | 4 | 5 | 5 | 5 | 5 | 5 | 5 |
| EP04BRT | Task 4: Auditory attention, total number of correct counts (max=10) | Male | <18 years | 175 | 4 | 8 | 9 | 9 | 10 | 10 | 10 | 10 | 10 |
|  |  | Male | 18+ | 91 | 7 | 9 | 9 | 10 | 10 | 10 | 10 | 10 | 10 |
|  |  | Female | <18 years | 256 | 7 | 8 | 9 | 9 | 10 | 10 | 10 | 10 | 10 |
|  |  | Female | 18+ | 177 | 7 | 8 | 9 | 10 | 10 | 10 | 10 | 10 | 10 |
| EP05BRT | Task 5: Total correct answers (max=10=5 trials with 2 forms) | Male | <18 years | 176 | 8 | 10 | 10 | 10 | 10 | 10 | 10 | 10 | 10 |
|  |  | Male | 18+ | 91 | 8 | 9 | 10 | 10 | 10 | 10 | 10 | 10 | 10 |
|  |  | Female | <18 years | 256 | 7 | 9 | 9 | 10 | 10 | 10 | 10 | 10 | 10 |
|  |  | Female | 18+ | 177 | 5 | 9 | 9 | 10 | 10 | 10 | 10 | 10 | 10 |
| EP05T1M | Task 5: Auditory attention, mean time (millisec) until 1st click (whatever the response) | Male | <18 years | 176 | 1755 | 1953 | 2125 | 2514 | 3060 | 3696.5 | 4483 | 4980 | 7393 |
|  |  | Male | 18+ | 91 | 1514 | 1972 | 2131 | 2509 | 3096 | 3851 | 4427 | 5182 | 6614 |
|  |  | Female | <18 years | 256 | 1866 | 2246 | 2402 | 2785.5 | 3505 | 4312.5 | 5410 | 5604 | 7553 |
|  |  | Female | 18+ | 177 | 1552 | 1951 | 2253 | 2600 | 3288 | 3859 | 4727 | 5557 | 7954 |
| EP06BRT | Task 6: Total number of correct answers for form recognition (max=10=5*2) | Male | <18 years | 174 | 8 | 9 | 10 | 10 | 10 | 10 | 10 | 10 | 10 |
|  |  | Male | 18+ | 91 | 9 | 9 | 10 | 10 | 10 | 10 | 10 | 10 | 10 |
|  |  | Female | <18 years | 256 | 8 | 9 | 9 | 10 | 10 | 10 | 10 | 10 | 10 |
|  |  | Female | 18+ | 177 | 8 | 9 | 9 | 10 | 10 | 10 | 10 | 10 | 10 |
| EP06T1M | Task 6: Workin memory, mean time (millisec) until 1st click (whatever the response) | Male | <18 years | 174 | 1526 | 1948 | 2072 | 2458 | 2950.5 | 3561 | 4487 | 4841 | 6964 |
|  |  | Male | 18+ | 91 | 1533 | 1792 | 2081 | 2337 | 2824 | 3738 | 4429 | 4666 | 12517 |
|  |  | Female | <18 years | 256 | 1763 | 2047 | 2191 | 2665 | 3262 | 4534 | 5990 | 7340 | 9738 |
|  |  | Female | 18+ | 177 | 1598 | 1876 | 2123 | 2574 | 3271 | 4097 | 4756 | 5778 | 8457 |
| EP07ET02YT | Task 7, level 2: Mean duration of answers (milliseconds) | Male | <18 years | 173 | 690 | 743 | 796 | 874 | 959 | 1025 | 1143 | 1228 | 1526 |
|  |  | Male | 18+ | 90 | 673 | 727 | 785.5 | 849 | 927 | 1036 | 1145.5 | 1205 | 1345 |
|  |  | Female | <18 years | 256 | 699 | 751 | 800 | 855.5 | 952 | 1051.5 | 1148 | 1259 | 1706 |
|  |  | Female | 18+ | 177 | 686 | 759 | 777 | 850 | 922 | 1016 | 1102 | 1171 | 1259 |
| EP07ET03BR | Task 7, level 3: Stroop proactive inference, number of correct answers | Male | <18 years | 173 | 9 | 12 | 14 | 16 | 18 | 20 | 21 | 22 | 23 |
|  |  | Male | 18+ | 90 | 11 | 15 | 16 | 17 | 18 | 20 | 21 | 22 | 23 |
|  |  | Female | <18 years | 256 | 4 | 13 | 15 | 17 | 18 | 20 | 21 | 22 | 23 |
|  |  | Female | 18+ | 177 | 4 | 13 | 15 | 17 | 19 | 20 | 21 | 22 | 23 |
| EP08BR | Task 8: Immediate name recall, number of names correctly recalled | Male | <18 years | 175 | 3 | 4 | 4 | 5 | 6 | 7 | 8 | 8 | 9 |
|  |  | Male | 18+ | 91 | 4 | 4 | 5 | 5 | 6 | 7 | 8 | 8 | 9 |
|  |  | Female | <18 years | 256 | 4 | 5 | 5 | 6 | 7 | 8 | 8 | 9 | 9 |
|  |  | Female | 18+ | 177 | 4 | 5 | 5 | 6 | 7 | 8 | 8 | 9 | 9 |
| EP10RE | Task 10: Spatial span, number of successes | Male | <18 years | 175 | 0 | 2 | 3 | 3 | 5 | 6 | 6 | 6 | 6 |
|  |  | Male | 18+ | 91 | 0 | 3 | 3 | 3 | 5 | 5 | 6 | 6 | 6 |
|  |  | Female | <18 years | 256 | 0 | 2 | 3 | 3 | 4 | 5 | 6 | 6 | 6 |
|  |  | Female | 18+ | 177 | 0 | 2 | 3 | 3 | 5 | 5 | 6 | 6 | 6 |
| EP10T | Task 10: Spatial span, mean time (milliseconds) | Male | <18 years | 175 | 880 | 1343.8 | 1422.5 | 1804 | 2224.8 | 2921.8 | 3704 | 4283.5 | 5571 |
|  |  | Male | 18+ | 91 | 719.5 | 1375.8 | 1530.7 | 1694.8 | 2158.3 | 3096.2 | 3839.5 | 4918.7 | 6994.3 |
|  |  | Female | <18 years | 256 | 909.8 | 1205.3 | 1412.7 | 1784.3 | 2300.5 | 2959.3 | 3486.2 | 4226 | 5509.6 |
|  |  | Female | 18+ | 177 | 521 | 1293.8 | 1375.7 | 1707 | 2215.5 | 2678.5 | 3494.8 | 3931.2 | 5849.2 |
| EP11BR | Task 11, geometric forms: No correct responses | Male | <18 years | 176 | 3 | 4 | 5 | 6 | 7 | 7 | 7 | 8 | 8 |
|  |  | Male | 18+ | 91 | 3 | 4 | 5 | 6 | 6 | 7 | 8 | 8 | 8 |
|  |  | Female | <18 years | 256 | 2 | 4 | 5 | 6 | 6 | 7 | 8 | 8 | 8 |
|  |  | Female | 18+ | 177 | 3 | 4 | 5 | 6 | 7 | 7 | 8 | 8 | 8 |
| EP11MY | Task 11, Mean time for correct responses (milliseconds) | Male | <18 years | 176 | 3277 | 3629 | 3918 | 4702 | 5508.5 | 6629 | 7779 | 8703 | 9933 |
|  |  | Male | 18+ | 91 | 3142 | 3287 | 3623 | 4470 | 5646 | 6613 | 7501 | 8376 | 11916 |
|  |  | Female | <18 years | 256 | 2930 | 3638 | 4070 | 4825.5 | 5638.5 | 6793 | 7988 | 8945 | 10792 |
|  |  | Female | 18+ | 177 | 3381 | 3732 | 4095 | 4735 | 5604 | 6315 | 7779 | 8303 | 9327 |
| EP12BR | Task 12: Phoneme comprehension, No of correct answers (max=10) | Male | <18 years | 176 | 7 | 7 | 8 | 8 | 9 | 9 | 9 | 9 | 9 |
|  |  | Male | 18+ | 91 | 7 | 7 | 8 | 8 | 9 | 9 | 9 | 9 | 9 |
|  |  | Female | <18 years | 256 | 7 | 8 | 8 | 8 | 9 | 9 | 9 | 9 | 9 |
|  |  | Female | 18+ | 177 | 7 | 8 | 8 | 8 | 9 | 9 | 9 | 9 | 9 |
| EP12MY | Task 12, word comprehension: mean time for correct response (milliseconds) | Male | <18 years | 176 | 829 | 1103 | 1157 | 1287 | 1433.5 | 1642 | 1913 | 2053 | 2452 |
|  |  | Male | 18+ | 91 | 955 | 1148 | 1218 | 1325 | 1452 | 1664 | 1874 | 2150 | 2769 |
|  |  | Female | <18 years | 256 | 943 | 1087 | 1138 | 1273 | 1426 | 1644 | 1840 | 1940 | 2263 |
|  |  | Female | 18+ | 177 | 970 | 1093 | 1126 | 1245 | 1379 | 1536 | 1733 | 1899 | 2198 |
| EP13BA_P | Task 13: Semantic associations, No correct associations(max=10) | Male | <18 years | 176 | 8 | 9 | 9 | 10 | 10 | 10 | 10 | 10 | 10 |
|  |  | Male | 18+ | 91 | 8 | 9 | 9 | 9 | 10 | 10 | 10 | 10 | 10 |
|  |  | Female | <18 years | 256 | 8 | 9 | 9 | 9 | 10 | 10 | 10 | 10 | 10 |
|  |  | Female | 18+ | 177 | 8 | 9 | 9 | 9 | 10 | 10 | 10 | 10 | 10 |
| EP13RDP | Task 13: Semantic associations, No trials over time limit | Male | <18 years | 176 | 0 | 0 | 0 | 0 | 0 | 0 | 0 | 0 | 1 |
|  |  | Male | 18+ | 91 | 0 | 0 | 0 | 0 | 0 | 0 | 0 | 0 | 1 |
|  |  | Female | <18 years | 256 | 0 | 0 | 0 | 0 | 0 | 0 | 0 | 1 | 1 |
|  |  | Female | 18+ | 177 | 0 | 0 | 0 | 0 | 0 | 0 | 0 | 0 | 1 |
| EP13RI | Task 13: Semantic associations, No of exact responses for naming | Male | <18 years | 176 | 9 | 9 | 10 | 10 | 10 | 10 | 10 | 10 | 10 |
|  |  | Male | 18+ | 91 | 8 | 10 | 10 | 10 | 10 | 10 | 10 | 10 | 10 |
|  |  | Female | <18 years | 256 | 8 | 9 | 9 | 10 | 10 | 10 | 10 | 10 | 10 |
|  |  | Female | 18+ | 177 | 8 | 9 | 10 | 10 | 10 | 10 | 10 | 10 | 10 |
| EP14BR_P | Task 14: Visiospatial logic (matrices), nb of correct answers | Male | <18 years | 175 | 2 | 6 | 7 | 8 | 9 | 11 | 12 | 13 | 14 |
|  |  | Male | 18+ | 91 | 3 | 6 | 7 | 8 | 9 | 11 | 12 | 13 | 15 |
|  |  | Female | <18 years | 256 | 3 | 5 | 6 | 7 | 9 | 10 | 11 | 12 | 13 |
|  |  | Female | 18+ | 177 | 2 | 7 | 7 | 8 | 10 | 11 | 12 | 13 | 14 |
| EP14TT | Task 14: Visiospatial logic (matrices), Total time | Male | <18 years | 175 | 84387 | 104287 | 114961 | 145826 | 186308 | 217060 | 258094 | 283564 | 349296 |
|  |  | Male | 18+ | 91 | 79550 | 106312 | 114869 | 134727 | 170101 | 207039 | 277068 | 327761 | 409062 |
|  |  | Female | <18 years | 256 | 80731 | 98493 | 111836 | 133287.5 | 175731.5 | 236157 | 286123 | 306610 | 351042 |
|  |  | Female | 18+ | 177 | 93197 | 106768 | 121270 | 142523 | 184105 | 238717 | 282815 | 309734 | 420742 |
| EP17ET01BR | Task 17, Delayed name recall, free recall: no of correct answers | Male | <18 years | 175 | 2 | 3 | 4 | 5 | 6 | 7 | 8 | 9 | 9 |
|  |  | Male | 18+ | 91 | 3 | 4 | 5 | 6 | 7 | 8 | 8 | 8 | 9 |
|  |  | Female | <18 years | 256 | 3 | 5 | 5 | 6 | 7 | 8 | 9 | 9 | 9 |
|  |  | Female | 18+ | 177 | 3 | 5 | 6 | 7 | 8 | 8 | 9 | 9 | 9 |
| EP17ET02BR | Task 17, Delayed name recall, cued recall: no of correct answers | Male | <18 years | 176 | 2 | 3 | 4 | 5 | 7 | 7 | 8 | 9 | 9 |
|  |  | Male | 18+ | 91 | 4 | 5 | 5 | 6 | 7 | 8 | 8 | 9 | 9 |
|  |  | Female | <18 years | 256 | 3 | 5 | 6 | 6 | 7 | 8 | 9 | 9 | 9 |
|  |  | Female | 18+ | 177 | 3 | 5 | 6 | 7 | 8 | 8 | 9 | 9 | 9 |
| EP18BN | Task 18, surname recognition: no. of names correctly recognized | Male | <18 years | 176 | 0 | 1 | 2 | 3 | 4 | 6 | 7 | 8 | 9 |
|  |  | Male | 18+ | 91 | 0 | 2 | 2 | 3 | 5 | 6 | 8 | 8 | 9 |
|  |  | Female | <18 years | 256 | 1 | 2 | 3 | 4 | 6 | 7 | 8 | 9 | 9 |
|  |  | Female | 18+ | 177 | 1 | 2 | 3 | 5 | 6 | 7 | 8 | 9 | 9 |
| EP19_20C | Tasks 19 and 20, verbal fluency, semantic phonemic cue: nb of correct answers 60s | Male | <18 years | 176 | 14 | 16 | 17 | 20 | 24 | 29.5 | 33 | 37 | 40 |
|  |  | Male | 18+ | 91 | 11 | 15 | 18 | 22 | 28 | 31 | 35 | 37 | 42 |
|  |  | Female | <18 years | 255 | 14 | 17 | 19 | 24 | 28 | 32 | 36 | 39 | 42 |
|  |  | Female | 18+ | 177 | 17 | 20 | 21 | 25 | 30 | 34 | 38 | 42 | 45 |
| EP21T | Task 21, narrative story: total nb of correct answers (max=27) | Male | <18 years | 176 | 1 | 6 | 8 | 11 | 14 | 17 | 19 | 21 | 23 |
|  |  | Male | 18+ | 91 | 5 | 7 | 8 | 11 | 13 | 17 | 20 | 22 | 24 |
|  |  | Female | <18 years | 256 | 3 | 6 | 8 | 11 | 14 | 17 | 19 | 21 | 24 |
|  |  | Female | 18+ | 177 | 5 | 8 | 9 | 13 | 15 | 18 | 20 | 21 | 26 |
| EP22T | Task 22, descriptive story: total nb of correct answers (max=27) | Male | <18 years | 176 | 4 | 6 | 7 | 9 | 12 | 15 | 18 | 21 | 23 |
|  |  | Male | 18+ | 91 | 0 | 6 | 8 | 10 | 14 | 16 | 18 | 21 | 24 |
|  |  | Female | <18 years | 256 | 5 | 6 | 8 | 10 | 13 | 17 | 20 | 21 | 23 |
|  |  | Female | 18+ | 177 | 5 | 9 | 10 | 12 | 15 | 17 | 21 | 22 | 24 |
| EP23BR | Task 23: Nb of correct answers (max=35) | Male | <18 years | 176 | 8 | 12 | 14 | 17 | 21 | 25 | 28 | 29 | 33 |
|  |  | Male | 18+ | 91 | 7 | 13 | 15 | 20 | 24 | 26 | 28 | 31 | 33 |
|  |  | Female | <18 years | 256 | 6 | 11 | 12 | 16 | 19 | 23.5 | 27 | 28 | 31 |
|  |  | Female | 18+ | 177 | 11 | 14 | 17 | 20 | 24 | 26 | 29 | 30 | 32 |
| EP23MR | Task 23: Vocabulary, no of incorrect answers | Male | <18 years | 176 | 2 | 6 | 7 | 10 | 14 | 17 | 20 | 23 | 27 |
|  |  | Male | 18+ | 91 | 2 | 4 | 7 | 9 | 11 | 14 | 19 | 22 | 26 |
|  |  | Female | <18 years | 256 | 4 | 7 | 8 | 11 | 15 | 19 | 23 | 24 | 26 |
|  |  | Female | 18+ | 177 | 3 | 5 | 6 | 9 | 11 | 15 | 18 | 20 | 23 |
| EP24DF | Task 24 implicit memory: difference | Male | <18 years | 176 | -0.2 | 0.2 | 0.2 | 0.6 | 1 | 1.4 | 1.8 | 2 | 3.4 |
|  |  | Male | 18+ | 91 | -0.2 | 0.2 | 0.4 | 0.6 | 1 | 1.4 | 1.8 | 2.4 | 2.6 |
|  |  | Female | <18 years | 256 | -0.4 | 0 | 0.2 | 0.6 | 1 | 1.4 | 1.8 | 2.2 | 3 |
|  |  | Female | 18+ | 177 | -1 | 0 | 0.2 | 0.6 | 1 | 1.4 | 1.8 | 2 | 2.4 |
| EP25T_P | Task 25: scoring the design | Male | <18 years | 146 | 47 | 55 | 61 | 68 | 73 | 77 | 80 | 80 | 80 |
|  |  | Male | 18+ | 80 | 39 | 45.5 | 62.5 | 67 | 72.5 | 77 | 79.5 | 80 | 80 |
|  |  | Female | <18 years | 232 | 46 | 55 | 62 | 68 | 72.5 | 77 | 80 | 80 | 80 |
|  |  | Female | 18+ | 149 | 51 | 55 | 62 | 70 | 74 | 77 | 80 | 80 | 80 |

**Supplementary Table 2:** Percentiles of COGNITO tasks by sex and education.
